# A Novel Porphyrin-Containing Polyimide Nanofibrous Membrane for Colorimetric and Fluorometric Detection of Pyridine Vapor

**DOI:** 10.3390/s131115758

**Published:** 2013-11-19

**Authors:** Yuanyuan Lv, Yani Zhang, Yanglong Du, Jiayao Xu, Junbo Wang

**Affiliations:** School of Medicine, Zhejiang University City College, Hangzhou 310015, China; E-Mails: zjlxzyn@sina.com (Y.Z.); duyl@zucc.edu.cn (Y.D.); zjxjy1992@163.com (J.X.)

**Keywords:** zinc porphyrin, nanofibrous membrane, colorimetric sensor, fluorometric sensor, pyridine vapor

## Abstract

A novel zinc porphyrin-containing polyimide (ZPCPI) nanofibrous membrane for rapid and reversible detection of trace amounts of pyridine vapor is described. The membrane displays a distinct color change, as well as dramatic variations in absorption and fluorescent emission spectra, upon exposure to pyridine vapor. This condition allows the detection of the analyte at concentrations as low as 0.041 ppm. The vapochromic and spectrophotometric responses of the membrane are attributed to the formation of the ZPCPI-pyridine complex upon axial coordination. From surface plasmon resonance analysis, the affinity constant of ZPCPI-pyridine complex was calculated to be (3.98 ± 0.25) × 10^4^ L·mol^−1^. The ZPCPI nanofibrous membrane also showed excellent selectivity for pyridine vapor over other common amines, confirming its applicability in the manufacture of pyridine-sensitive gas sensors.

## 1. Introduction

The detection of gaseous pollutants using sensitive and reversible sensors has attracted considerable attention in recent years. Among a variety of new analytical tools under development, optochemical gas sensors are especially attractive because of their sensitivity and simplicity [1–9]. They usually contain a chemically active sensing agent that exhibits efficient changes in some photophysical property (e.g., color, fluorescence, λ_max_ shift of the UV-vis spectrum, *etc.*) upon exposure to an analyte. The choice of indicator (probe) is obviously the key step in the design of an optochemical gas sensor.

Metalloporphyrins are potentially attractive chromogenic indicators for the development of novel optical sensors because of the open coordination sites in these indicators for axial ligation, large spectral shifts upon ligand binding, and intense coloration. Several studies have reported on the successful application of metalloporphyrins in optical devices, generally related to the detection of O_2_, Cl_2_, CO, HCl, and alcohols [10–16]. However, extensive studies on metalloporphyrin-based gas sensors are still required to improve their sensitivity, selectivity, and response-reversal time. Moreover, sensor stability should also be considered in developing special gas sensors intended for use in harsh environments.

**Figure 1.**
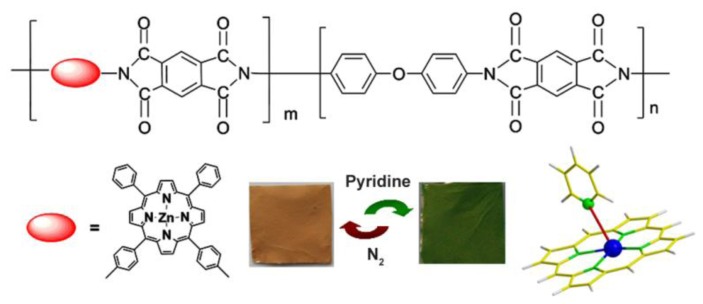
Molecular structure of ZPCPI used in this study and the schematic illustration for the whole vapochromic and spectrophotometric detection of pyridine vapor.

In this study, we report the first attempt to apply a vapochromic and spectrophotometric zinc porphyrin-containing polyimide (ZPCPI) nanofibrous membrane to the detection of trace amounts of pyridine vapor. The molecular structure of ZPCPI is illustrated in Figure 1.

Pyridine is widely used as an organic reagent in various industrial processes. This substance is harmful if inhaled, ingested, or absorbed by the skin because of its toxicity and carcinogenicity [17–19], therefore, a sensitive and rapid detection of pyridine vapor is valuable in industrial and environmental monitoring. Zinc porphyrin was chosen for this study because of its strong affinity with nitrogen ligands to form a five-coordinated complex, which results in measurable changes in its photophysical properties [20–24]. Incorporation of zinc porphyrin fluorophore into a polyimide backbone facilitates pyridine vapor detection in harsh environments because of the mechanical stability, as well as the high thermal and chemical resistance, of polyimide. Moreover, an electrospun nanofibrous polymer membrane with large available surface area and high porosity has the potential to provide extremely high sensitivity and fast response time for sensing applications [25–30]. Therefore, the feasibility for a sensitive, selective, and rapid detection of pyridine vapor using ZPCPI nanofibrous membrane was extensively investigated. As an insight into the affinity interactions between the nanofibrous membrane of ZPCPI and pyridine at molecular level, quantitative analysis was carried out with surface plasmon resonance (SPR). Adsorption isotherms were analyzed to determine the sensitivity of pyridine binding to the nanofibrous membrane of ZPCPI.

## 2. Experimental Section

### 2.1. Materials

Zinc 5,10-bis (4-aminophenyl)-15,20-diphenylporphyrin (*cis*-ZnDATPP, ZP) was synthesized using a reported procedure [31,32]. Pyromellitic dianhydride (PMDA) and oxydianiline (ODA) were commercially obtained from Shanghai Chemical Agent Co. (Shanghai, China) and purified through sublimation above their gasification temperatures before use. *N*,*N*'-Dimethylacetamide (DMAc, analytical reagent grade, purchased from Shanghai Chemical Agent Co.), was dried over 4 Å molecular sieves before use. All other reagents were analytical grade and used as received without further purification.

### 2.2. Preparation of ZPCPI Nanofibrous Membrane

For polymer synthesis, *cis*-ZnDATPP was copolymerized into polyimide backbones with PMDA and ODA to obtain copoly (amic acid) (ZPPAA), “precursors” of the expected zinc porphyrin-containing polyimide (ZPCPI) [33]. A typical procedure for the electrospinning of ZPCPI nanofibrous membrane is as described in the literature [33–36]. ZPPAA was dissolved in DMAc to form a 16 wt.% solution. The resulting viscous solution was pumped through a metal syringe needle at a constant rate of 1.0 mL·h^−1^ using a microinfusion pump (WZ-50C2, Zhejiang University Medical Instrument Co., LTD, Hangzhou, China). To prepare ZPCPI nanofibers, imidization of the as-spun ZPPAA nanofibers was performed by heating step by step under N_2_ atmosphere at 80 °C (0.5 h), 160 °C (1 h) and 250 °C (4 h). Figure 1 illustrates the molecular structure of the target polymers.

### 2.3. Apparatus

The inherent viscosity was measured using an Ubbelohde viscometer (Midwest, Shanghai, China). The ^1^H-NMR spectrum was obtained in DMSO-*d_6_* on a Bruker Advance DMX500 NMR spectrometer (Bruker-Franzen Analytik GmbH, Bremen, Germany). Fluorescence emission spectra were obtained using a Shimadzu RF-5301 PC (Shimadzu, Kyoto, Japan) fluorescence spectrophotometer with a solid assembly to have excitation and emission at 45° to the membrane surface and samples were excited at 420 nm. UV-vis spectra were obtained in a quartz cell using Shimadzu UV 2450 spectrophotometer. UV-vis spectra in solid state were obtained using a Shimadzu integrating sphere assembly attached to the Shimadzu UV 2450 spectrophotometer. FESEM images were obtained on a field emission scanning electron microscope (FESEM, SIRION, FEI, Hillsboro, OR, USA) after the samples were sputter coated with 15 nm Au layer to make them conductive. All fluorescent images were obtained using confocal laser scanning microscopy (CLSM) with a Leica TCS SP5 confocal setup mounted on a Leica DMI 6000 CS inverted microscope (Leica Microsystems, Wetzlar, Germany) and was operated under the Leica Application Suite Advanced Fluorescence program. The excitation wavelength was 488 nm.

### 2.4. Trace Pyridine Vapor Sensing Performance of ZPCPI Nanofibrous Membrane

A sensor sample was prepared by depositing ZPCPI nanofibrous membrane on a clean glass cover slide that was placed in a sealed testing chamber to investigate the sensing performance of the ZPCPI nanofibrous membrane in the presence of trace amounts of pyridine vapor. The detection chamber was purged before the experiment with high purity (99.99%) N_2_, and a small volume of liquid pyridine was injected into the chamber and vaporized. The vapor concentration was calculated using the ideal gas law as:

(1) $$c=\frac{22.4\rho TV_{s}}{273.15MV}\times 10^{3}$$

where *c* is the concentration in ppm, *ρ* the density of the liquid sample in g·mL^−1^, *T* the temperature of the detection chamber in Kelvin, *V_s_* the volume of the liquid sample in μL, *M* the molecular weight of pyridine in g·mol^−1^, and *V* is the chamber volume in L (the testing chamber had a volume of 0.5 L [37]. The pyridine vapor sensing properties were demonstrated by the variation in the absorbance spectrum, fluorescence intensity, and color pattern developed on the sensor before or after exposure to pyridine vapor with pre-determined concentration for a certain time. After each measurement, the membrane was recovered by repeated puffing with high purity N_2_ before the next measurement, and the chamber was also purged again with N_2_.

### 2.5. Surface Plasmon Resonance (SPR) Analysis of the Interactions between ZPCPI and Pyridine

SPR measurements were performed on a Reichert SR7000 DC instrument (Reichert, Depew, NY, USA). The SPR chip was cleaned by dipping it in ethanol for 10 min, and then in a freshly made piranha solution (concentrated H_2_SO_4_ and 30% H_2_O_2_ with 3:1 proportion) for 1 min, followed by extensive rinsing with ultra-pure water (18.2 MΩ·cm). The chip was then dried in N_2_. ZPCPI nanofibrous membrane was electrospun on the SPR chip using the same electrospinning method previously mentioned. A certain concentration of pyridine solution was injected and allowed to flow over the sensory chip surface at a rate of 25 μL·min^−1^. Ultra-pure water was used as a buffer solution during the whole analysis process. Temperature was extensively controlled at 25.0 °C ± 0.1 °C throughout the experiment.

## 3. Results and Discussion

### 3.1. Preparation and Characterization of ZPCPI Nanofibrous Membrane

**Figure 2.**
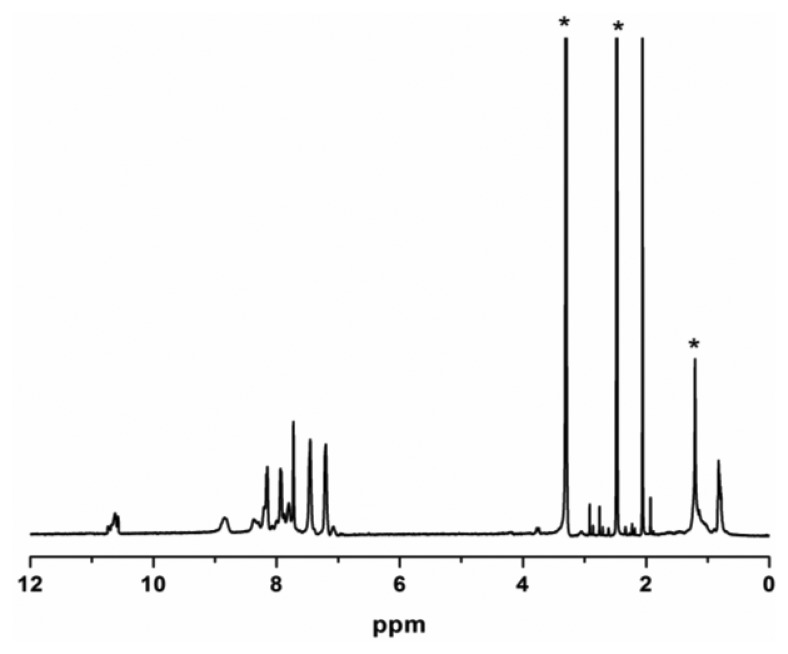
^1^H-NMR spectrum of ZPPAA in DMSO-*d*_6_. (*) indicates solvent impurity.

The molar ratios of ZP to ODA in the synthesized ZPPAA was 0.091 (calculated from the ^1^H-NMR Figure 2), which is very close to 0.10 (molar ratios of ZP to ODA in feed for ZPPAA synthesis), indicating a high conversion of porphyrin monomer. The inherent viscosity of the resulting ZPPAA was 0.433 dL·g^−1^, which was measured at a concentration of 0.5 g·dL^−1^ in DMAc at 30 °C using an Ubbelohde viscometer. A nanofibrous membrane was successfully prepared by electrospinning a 16 wt.% ZPPAA solution in DMAc and ZPPAA nanofibers were converted into ZPCPI nanofibers through the imidization process.

**Figure 3.**
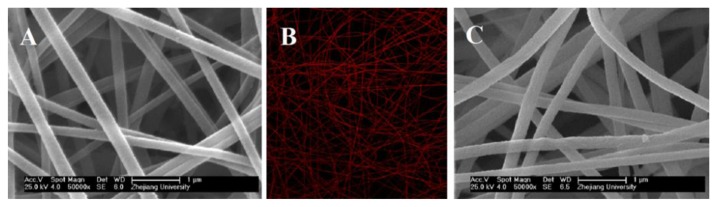
FESEM micrograph (**A**) and CLSM image (**B**) (×40) of the ZPCPI nanofibrous membrane (*λ_ex_* = 488 nm); FESEM micrograph (**C**) of the ZPCPI nanofibrous membrane after consecutively used for five times.

**Figure 4.**
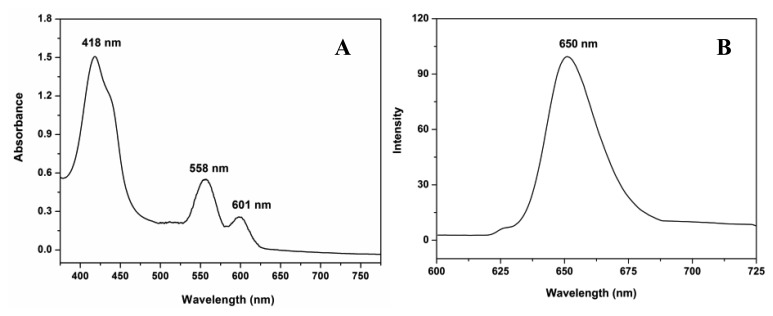
Absorption (**A**) and fluorescence emission (**B**) spectra of the ZPCPI nanofibrous membrane.

The FESEM micrographs indicate that smooth and uniform nanofibers with a diameter of approximately 295 nm ± 22 nm can be fabricated (Figure 3A). The absorption spectrum of the ZPCPI nanofibrous membrane displays the characteristic transitions of ZP with an intense Soret band at 418 nm and two weak Q-bands at 558 nm and 601 nm (Figure 4A). In agreement with this result, the ZPCPI nanofibrous membrane shows a distinct fluorescence emission spectrum of ZP when excited at 420 nm (Figure 4B). The CLSM image also visualized this fluorescence property. Red light was uniformly emitted from the nanofibrous membrane (Figure 3B).

### 3.2. Trace Pyridine Vapor Sensing Properties of ZPCPI Nanofibrous Membrane

**Figure 5.**
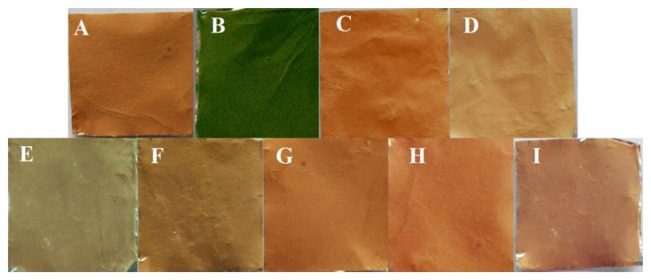
Photographs of the ZPCPI nanofibrous membrane when exposed to different vapors (30 ppm). (**A**) Blank; (**B**) Pyridine; (**C**) DEA; (**D**) TEA; (**E**) Py; (**F**) CA; (**G**) CO_2_; (**H**) H_2_O; and (**I**) recovered when puffed with N_2_.

Metalloporphyrins are a natural choice for colorimetric sensing applications because of their distinct color changes induced upon target gas binding [22,38,39]. Therefore, the evaluation of the colorimetric response behavior of ZPCPI nanofibrous membrane on pyridine vapor is necessary. As shown in Figure 5A,B, when exposed to pyridine vapor at room temperature (25 °C), an immediate and obvious color change from reddish brown to olivine was observed, indicating that the ZPCPI nanofibrous membrane can serve as a sensitive “naked-eye” indicator for pyridine vapor.

**Figure 6.**
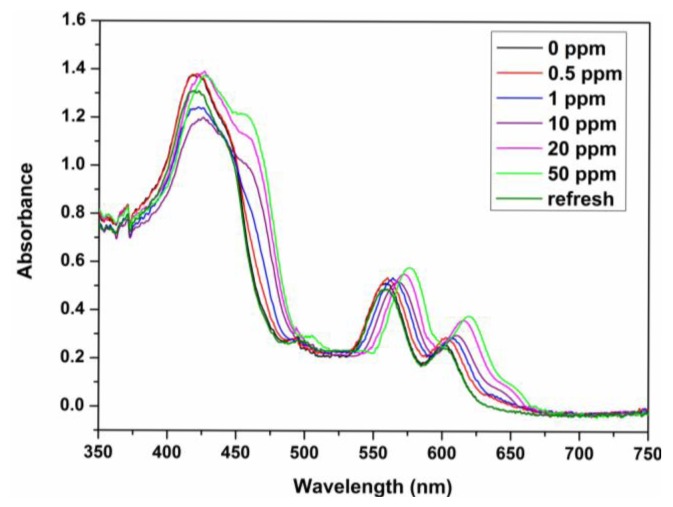
Absorption spectra of the ZPCPI nanofibrous membrane when exposed to different concentrations of pyridine vapor or recovered when puffed with N_2_.

**Figure 7.**
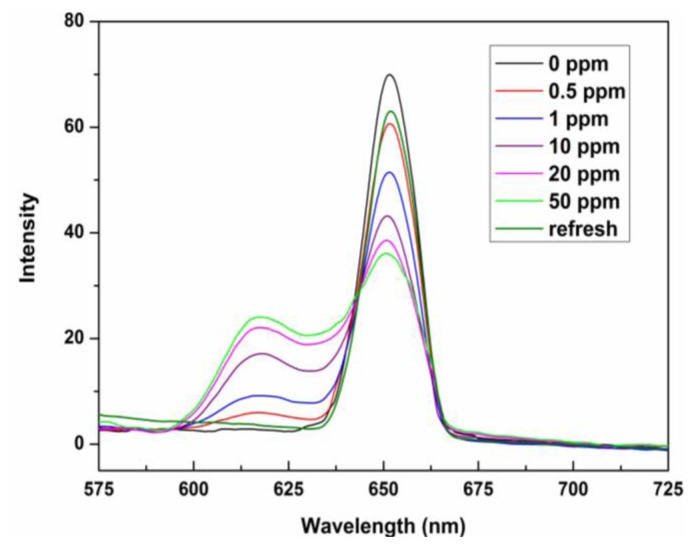
Fluorescence emission spectra of the ZPCPI nanofibrous membrane when exposed to different concentrations of pyridine vapor or recovered when puffed with N_2_ (*λ_ex_* = 420 nm).

The sensing properties of ZPCPI nanofibrous membrane to pyridine vapor was further examined by solid state absorbance spectra to understand better the color variation of the sensing membrane. Although there were little jagged and truncated peaks in the Soret band due to the inevitable optical density effect of the testing membrane, a gradual bathochromic shift of Soret band from 418 nm to 445 nm could still be clearly observed when exposing the membrane to an increasing concentration of pyridine vapor (Figure 6). Meanwhile, the two Q-bands also red-shifted, and a new band appeared at 651 nm. Previous studies have reported that the four-coordinated ZP may accept only one axial nitrogen ligand to form five-coordinated complex, inducing a shift in absorption wavelength of ZP because of the internal charge transfer process (Figure 1). Moreover, ligation of nitrogen ligand at the fifth coordination site of the zinc ion altered the molecular planarity of ZP by pulling the metal ion slightly out of the porphyrin ring, which was responsible for the intensity fluctuations and appearance of new bands in the UV-vis spectrum [15,16,20,21,24,40]. As a result, these colorimetric and spectroscopic responses are attributed to the electronic state changes and geometrical distortion of porphyrin through strong pyridine coordination. Concomitantly, a decrease in fluorescence intensities, centered at 650 nm of the ZPCPI nanofibrous membrane, was observed, and a new broad emission band appeared between 600 nm and 625 nm as the concentrations of pyridine vapor increased (Figure 7). The new peak at 617 nm also confirmed the formation of the ZPCPI-pyridine complex. Plotting pyridine vapor concentrations against absorbance variation (*ΔA*), which refers to the difference between the absorbance intensities of the ZPCPI nanofibrous membrane at 445 nm before and after exposure to different pyridine vapor concentrations, yielded the following equation [41]:

(2) Absorbance=0.0678×concentration+0.0086

(Correlation coefficient = 0.9989). In addition, the calculated detection limit of the solid state sensor was found to be 0.041 ppm.

The contacted membrane can be regenerated and reused for many times. The color of the ZPCPI nanofibrous membrane returned to its original reddish brown color after the membrane was repeatedly puffed with N_2_ for only 3 min (Figure 5I). At the same time, the absorbance and emission spectra nearly reverted back to their original values (Figures 6 and 7).

The chemical and mechanical stability of polyimide allowed the reversible, stable, and reusable pyridine vapor detection of the nanoporous membrane without any structural damage even after five regeneration cycles (Figure 3C). The reversible and reusable feature of the nanosensor membrane overcomes disposal problems, and combined with its small size, it may be used as a portable detector for pyridine vapor sensing.

### 3.3. Selectivity

**Figure 8.**
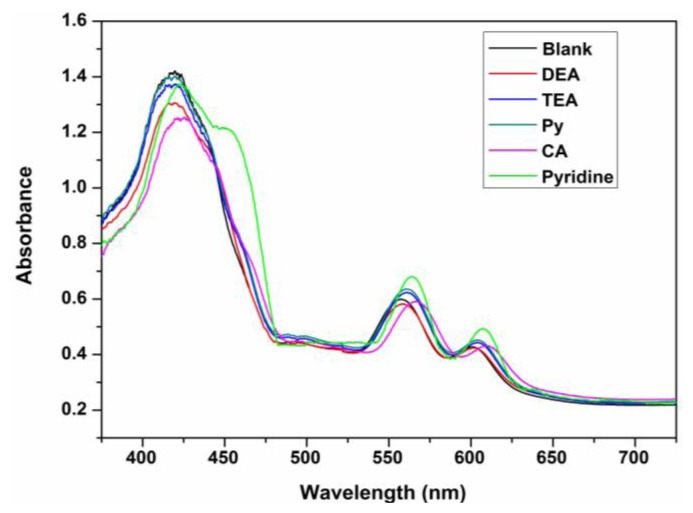
Absorption spectra of the ZPCPI nanofibrous membrane exposed to different vapors (*λ_ex_* = 420 nm).

Excellent chemosensors require high selectivity. Here, we first investigated the coloration responses of common amines, including dimethylamine (DEA), triethylamine (TEA), which have branched molecular structures, and pyrrole (Py) and cyclohexylamine (CA), which have cyclic molecular structures and other potential interfering gas-phase species (e.g., CO_2_ and H_2_O) to ZPCPI nanofibrous membrane. No significant color changes were observed in the parallel experiments, except for pyridine vapor (Figure 5A–H). These findings confirm that the ZPCPI nanofibrous membrane can be used as a selective colorimetric sensing material for pyridine vapor. Moreover, the selectivity of the nanofibrous membrane was further studied by UV-vis measurements. Flushing the sensor with DEA, TEA, Py, CA vapor in ppm concentrations exhibited little effects on the sensor response compared with pyridine vapor (Figure 8). The small response of ZPCPI nanofibrous membrane for DEA, TEA and CA may be due to the steric effects of these branched amines compared with pyridine. Besides, a π-π stacking interaction between porphyrin ring and the pyridine molecule can occur to form a stable structure. As for Py which has the same cyclic structure as pyridine, the relative less response could be ascribed to the basicity. In solution the pyridine has the modest *pK_a_* while in the gas phase the pyridine is the most basic amine among those amines listed in our study. As a result, we can conclude that both the strong gas phase basicity and the small steric hindrance of pyridine allows it to interact easily with the ZP dye, thus forming a stable complex compared with other amines [42,43].

### 3.4. Stoichiometric and Affinity Constant of ZPCPI-Pyridine Complex

**Figure 9.**
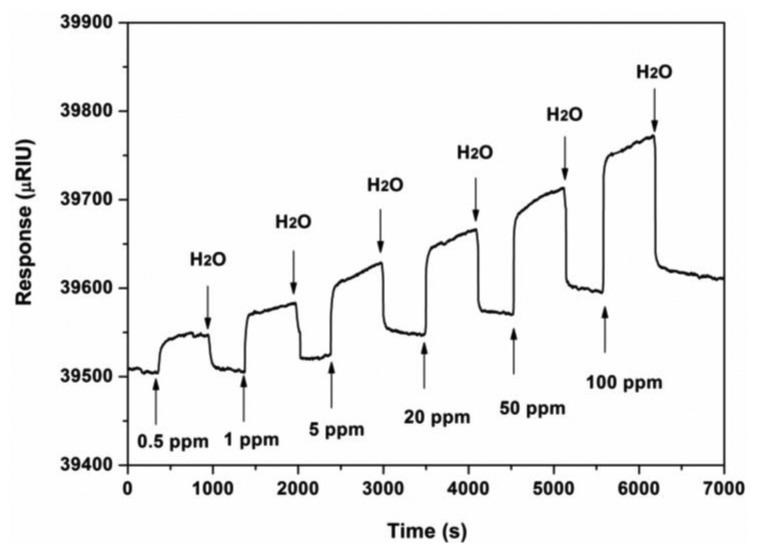
SPR response induced by the ZPCPI nanofibrous membrane deposited on an SPR chip when exposed to pyridine solution with different concentrations. H_2_O is the buffer solution throughout the whole process.

To assess further the affinity interactions between ZPCPI nanofibrous membrane and pyridine in a solution at the molecular level, an SPR analysis was performed in this study. The typical SPR responses of the ZPCPI nanofibrous membrane deposited on the SPR chip surface to the pyridine aqueous solution with different concentrations are shown in Figure 9. Increasing pyridine concentration leads to a progressive increase of SPR response. The nanofibrous membrane was then regenerated by rinsing with ultra-pure water. The apparent binding affinity constant (*K_a_*) of ZPCPI nanofibrous membrane with pyridine is found to be (3.98 ± 0.25) × 10^4^ L·mol^−1^ using a Langmuir adsorption model equation [44]. This result indicates that the ZPCPI nanofibrous membrane has a great potential as a sensory material for pyridine.

## 4. Conclusions

In conclusion, a novel ZPCPI nanofibrous membrane has been fabricated as a sensitive, selective, and reversible optochemical gas sensor. Pyridine detection using the proposed sensor can be performed using the naked eye, UV-vis spectrophotometry, and fluorescence variations, with a detection limit as low as 0.041 ppm. These photophysical responses of the sensing membrane are attributed to the electronic state changes and geometrical distortion of ZP via strong pyridine coordination. Given the small molecular size and aromatic nature as well as the strong gas phase basicity of pyridine, the selectivity of the sensor for pyridine vapor is excellent compared with other common amines and potential interfering gas-phase species. The apparent binding affinity constant calculated from the SPR analysis also confirms the distinct sensitivity of the ZPCPI nanofibrous membrane sensor toward pyridine. Moreover, the superior chemical and mechanical stability of the nanofibrous membrane sensor makes it promising for monitoring pyridine vapor in harsh environments without any structural damage. Significantly, although the proposed method was intended for pyridine detection, the design strategy is general enough to be readily extended to the development of chemosensors for a variety of other species, by choosing porphyrins with appropriate metal ion centers. Efforts to extend this design principle for the detection of different analytes are currently underway in our laboratory.

## References

[1] Xie Y.Z., Shan G.G., Zhou Z.Y., Su Z.M. (2013). Schiff-base as highly sensitive and reversible chemosensors for HCl gas. Sens. Actuators B: Chem..

[2] Gonçalves V.C., Balogh D.T. (2012). Optical chemical sensors using polythiophene derivatives as active layer for detection of volatile organic compounds. Sens. Actuators B: Chem..

[3] Lee J., Seo S., Kim J. (2012). Colorimetric detection of warfare gases by polydiacetylenes toward equipment-free detection. Adv. Funct. Mater..

[4] Spadavecchia J., Rella R., Siciliano P., Manera M.G., Alimelli A., Paolesse R., di Natale C., D'Amico A. (2006). Optochemical vapour detection using spin coated thin film of ZnTPP. Sens. Actuators B: Chem..

[5] Tan Q.L., Pei X.D., Zhu S.M., Sun D., Liu J., Xue C.H., Liang T., Zhang W.D., Xiong J.J. (2013). Development of an optical gas leak sensor for detecting ethylene, dimethyl ether and methane. Sensors.

[6] Chao D.M., Jia X.T., Bai F.Q., Liu H.T., Cui L.L., Berda E.B., Wang C. (2012). An efficient fluorescent sensor for redox active species based on novel poly (aryl ether) containing electroactive pendant. J. Mater. Chem..

[7] Peng H.J., Cheng Y.F., Dai C.F., King A.L., Predmore B.L., Lefer D.J., Wang B.H. (2011). A fluorescent probe for fast and quantitative detection of hydrogen sulfide in blood. Angew. Chem. Int. Ed..

[8] Liu C.R., Pan J., Li S., Zhao Y., Wu L.Y., Berkman C.E., Whorton A.R., Xian M. (2011). Capture and visualization of hydrogen sulfide by a fluorescent probe. Angew. Chem. Int. Ed..

[9] Tang Z.L., Yang J.H., Yu J.Y., Cui B. (2010). A colorimetric sensor for qualitative discrimination and quantitative detection of volatile amines. Sensors.

[10] Rakow N.A., Suslick K.S. (2000). A colorimetric sensor array for odour visualization. Nature.

[11] Giancane G., Valli L. (2012). State of art in porphyrin Langmuir–Blodgett films as chemical sensors. Adv. Colloid Interface Sci..

[12] Di Natale C., Paolesse R., D'Amico A. (2007). Metalloporphyrins based artificial olfactory receptors. Sens. Actuators B: Chem..

[13] Di Natale C., Monti D., Paolesse R. (2010). Chemical sensitivity of porphyrin assemblies. Mater. Today.

[14] Zhang C., Suslick K.S. (2005). A colorimetric sensor array for organics in water. J. Am. Chem. Soc..

[15] Dunbar A.D.F., Richardson T.H., McNaughton A.J., Hutchinson J., Hunter C.A. (2006). Investigation of free base, Mg, Sn, and Zn substituted porphyrin LB films as gas sensors for organic analytes. J. Phys. Chem. B.

[16] Shirsat M.D., Sarkar T., Kakoullis J., Myung N.V., Konnanath B., Spanias A., Mulchandani A. (2012). Porphyrin-functionalized single-walled carbon nanotube chemiresistive sensor arrays for VOCs. J. Phys. Chem. C.

[17] Singh K.P., Basant N., Malik A., Singh V.K., Mohan D. (2008). Chemometrics assisted spectrophotometric determination of pyridine in water and wastewater. Anal. Chim. Acta.

[18] Lv B.Q., Cheng C.M., Mo Y., Yuan H.Y., Xiao D. (2008). A simple strategy for pyridine visual sensing by the in-situ formation of tetranuclear copper iodine pyridine microcrystalline film on copper foil. Thin Solid Film..

[19] Elosua C., Bariain C., Matias I.R., Rodriguez A., Colacio E., Salinas-Castillo A., Segura-Carretero A., Fernandez-Gutiérrez A. (2008). Pyridine vapors detection by an optical fibre sensor. Sensors.

[20] Takulapalli B.R., Laws G.M., Liddell P.A., Andrasson J., Erno Z., Gust D., Thornton T.J. (2008). Electrical detection of amine ligation to a metalloporphyrin via a hybrid SOI-MOSFET. J. Am. Chem. Soc..

[21] Zhang Y., Yang R.H., Liu F., Li K.A. (2004). Fluorescent sensor for imidazole derivatives based on monomer-dimer equilibrium of a zinc porphyrin complex in a polymeric film. Anal. Chem..

[22] Gulino A., Bazzano S., Mineo P., Scamporrino E., Vitalini D., Fragalà I. (2005). Characterization, optical recognition behavior, sensitivity, and selectivity of silica surfaces functionalized with a porphyrin monolayer. Chem. Mater..

[23] Kirksey C.H., Hambright P., Storm C.B. (1969). Stability constants and proton magnetic resonance studies of zinc α,β,γ,δ-tetraphenylporphin and substituted pyridines. Inorg. Chem..

[24] Paske A.C., Earl L.D., O'Donnell J.L. (2011). Interfacially polymerized metalloporphyrin thin films for colorimetric sensing of organic vapors. Sens. Actuator. B: Chem..

[25] Chigome S., Torto N. (2011). A review of opportunities for electrospun nanofibers in analytical chemistry. Anal. Chim. Acta.

[26] Li Z.Y., Zhang H.N., Zheng W., Wang W., Huang H.M., Wang C., MacDiarmid A.G., Wei Y. (2008). Highly sensitive and stable humidity nanosensors based on LiCl doped TiO_2_ electrospun nanofibers. J. Am. Chem. Soc..

[27] Song X.F., Wang Z.J., Liu Y.B., Wang C., Li L.J. (2009). A highly sensitive ethanol sensor based on mesoporous ZnO-SnO_2_ nanofibers. Nanotechnology.

[28] Agarwal S., Wendorff J.H., Greiner A. (2008). Use of electrospinning technique for biomedical applications. Polymer.

[29] Lin Q.Q., Li Y., Yang M.J. (2012). Polyaniline nanofiber humidity sensor prepared by electrospinning. Sens. Actuators B: Chem..

[30] Lin Q.Q., Li Y., Yang M.J. (2012). Highly sensitive and ultrafast response surface acoustic wave humidity sensor based on electrospun polyaniline/poly (vinyl butyral) nanofibers. Anal. Chim. Acta.

[31] Luguya R., Jaquinod L., Fronczek F.R., Vicente M.G.H., Smith K.M. (2004). Synthesis and reactions of *meso*-(*p*-nitrophenyl) porphyrins. Tetrahedron.

[32] Anannarukan W., Tantayanon S., Zhang D., Alemán E.A., Modarelli D.A., Harris F.W. (2006). Soluble polyimides containing trans-diaminotetraphenylporphyrin: Synthesis and photoinduced electron transfer. Polymer.

[33] Lv Y.Y., Wu J., Wan L.S., Xu Z.K. (2008). Novel porphyrinated polyimide nanofibers by electrospinning. J. Phys. Chem. C.

[34] Lv Y.Y., Wu J., Xu Z.K. (2010). Colorimetric and fluorescent sensor constructing from the nanofibrous membrane of porphyrinated polyimide for the detection of hydrogen chloride gas. Sens. Actuator. B: Chem..

[35] Che A.F., Liu Z.M., Huang X.J., Wang Z.G., Xu Z.K. (2008). Chitosan-modified poly (acrylonitrile-co-acrylic acid) nanofibrous membranes for the immobilization of concanavalin A. Biomacromolecules.

[36] Wan L.S., Ke B.B., Xu Z.K. (2008). Electrospun nanofibrous membranes filled with carbon nanotubes for redox enzyme immobilization. Enzyme Microb. Technol..

[37] Wang X.F., Si Y., Wang J.L., Ding B., Yu J.Y., Al-Deyab S.S. (2012). A facile and highly sensitive colorimetric sensor for the detection of formaldehyde based on electro-spinning/netting nano-fiber/nets. Sens. Actuators B: Chem..

[38] Johnson-White B., Zeinali M., Shaffer K.M., Patterson C.H., Charles P.T., Markowitz M.A. (2007). Detection of organics using porphyrin embedded nanoporous organosilicas. Biosens. Bioelectron..

[39] Long J., Xu J.H., Yang Y.J., Wen J.F., Jia C.Y. (2011). A colorimetric array of metalloporphyrin derivatives for the detection of volatile organic compounds. Mater. Sci. Eng. B.

[40] Qin W., Parzuchowski P., Zhang W., Meyerhoff M.E. (2003). Optical sensor for amine vapors based on dimer-monomer equilibrium of indium (III) octaethylporphyrin in a polymeric film. Anal. Chem..

[41] Kalimuthu P., John S.A. (2008). Optochemical sensing of hydrogen chloride gas using meso-tetramesitylporphyrin deposited glass plate. Anal. Chim. Acta.

[42] Oberg K.I., Hodyss R., Beauchamp J.L. (2006). Simple optical sensor for amine vapors based on dyed silica microspheres. Sens. Actuators B: Chem..

[43] Hunter E.P.L., Lias S.G. (1998). Evaluated gas phase basicities and proton affinities of molecules: An update. J. Phys. Chem. Ref. Data.

[44] Zhang Y., Luo S.Z., Tang Y.J., Yu L., Hou K.Y., Cheng J.P., Zeng X.Q., Wang P.G. (2006). Carbohydrate-protein interactions by “clicked” carbohydrate self-assembled monolayers. Anal. Chem..

